# The impact of perioperative glucose variability on outcomes after hip fracture

**DOI:** 10.1097/MD.0000000000028728

**Published:** 2022-01-28

**Authors:** Anhua Long, Zongyan Xie, Xuefei Wang, Yakui Zhang, Dacheng Han

**Affiliations:** a Department of Orthopaedics, Beijing Luhe Hospital, Capital Medical University, Beijing, P.R. China; b Department of Clinical Pharmacology, Beijing Luhe Hospital, Capital Medical University, Beijing, P.R. China.

**Keywords:** diabetes mellitus, glucose variability, hip fracture, perioperative management

## Abstract

Diabetes is considered an independent risk factor for hip fracture. In the present study, we evaluated whether perioperative glucose variability (GV) was a significant predictor of the outcomes of patients with diabetes after hip fracture.

We analyzed the characteristics and outcomes of all patients with hip fractures admitted to our hospital between September 2008 and December 2012. Patients with diabetes were grouped into tertiles for GV, and multivariate survival analysis included age, sex, fracture type, mean fasting plasma glucose, and GV.

Among the 1099 patients included in this study, 239 (21.7%) had diabetes. Patients with diabetes were more likely to develop infectious complications (5.4% vs 2.8%, *P* = .045), and experience mortality postoperatively (1 month: 5.5% vs 2.7%, *P* = .052; 12 months: 15.1% vs 8.7%, *P* = .006). The postoperative mortality rate was increased across the GV tertiles, and GV was an independent predictor of 1- and 12-month mortality after surgery.

Patients with diabetes had poor prognoses after hip fracture. Perioperative GV is an independent predictor of mortality in patients with diabetes. Therefore, GV might be considered a valid additional parameter to consider in the management of these patients.

## 1 Introduction

It is generally accepted that diabetes mellitus is an independent risk factor for mortality and morbidity following the surgical repair of hip fracture.^[1–6]^ Patients with diabetes usually experience more complications,^[7,8]^ and they have a prolonged hospital length of stay (LOS)^[9]^; however, whether diabetes increases the risk of mortality remains controversial.^[3,10]^ Patients with diabetes may benefit from reasonable blood glucose control because some studies revealed that good perioperative glucose control reduced the risk of in-hospital death and shortened LOS.^[11–13]^ However, there is no gold standard of strict pre-operative glycemic control among patients with hip fracture.

Glucose variability (GV), which was believed to be associated with oxidative stress and to be more detrimental than hyperglycemia, has been suggested as a new target in blood glucose control.^[14–16]^ However, there is insufficient evidence to support its use in the surgery department.^[17]^ Could GV predict reasonable glucose control or a better clinical outcome? The aim of this study was to describe and examine the impact of GV on outcomes after hip fracture surgery.

## 2 Methods

All patients admitted to our hospital with a hip fracture were entered into the Hip Fracture Database. Patients included in this study fulfilled the criteria of age of at least 60 years and having sustained a femoral neck or intertrochanteric fracture. Admitted patients who did not undergo an operation for various reasons were excluded from this study. The study protocol was approved by the Ethics Committee of Beijing Luhe Hospital Affiliated to Capital Medical University. Because of the retrospective nature of the study and the fact that the patient data were anonymous, informed consent was not requested from the patients.

The collected data included demographics, the pre-fracture medical status, and nursing data. The demographic data and pre-morbidity condition were identified for each patient at the time of admission. The nursing records were created by the nurses while peripheral blood glucose (usually from the fingers) was checked. All of these data were collected retrospectively by chart abstraction according to our hospital's Electronic Medical Record System.

We defined patients with diabetes as those admitted with a diagnosis of diabetes that was controlled by diet or diabetic medication and those who were diagnosed by endocrinological consultation with doctors during hospitalization. Diabetes may be diagnosed based on A1c criteria or plasma glucose criteria, either the fasting plasma glucose (FPG) or the 2-hour plasma glucose value after a 75-g oral glucose tolerance test.

Patients were treated under the advice of the endocrinological consulting doctors pre-operatively. We obtained these patients’ 7-point glucose levels, as recorded by the nurses, to calculate their GV. Then, we calculated the coefficient of variation (CV) of FPG measurements, which is reported as being the easiest method of obtaining GV in clinical practice.^[18]^ Although it was possible to miss some peaks or nadirs using this GV calculation method, CV was acceptable because it was often impossible to obtain continuous glucose measurement data for each patient.

The comorbidities included cardiovascular disease (hypertension, coronary heart disease, myocardial infarction, cardiac insufficiency), cerebrovascular disease (cerebral infarction, encephalorrhagia, or stroke), respiratory disease (pre-existing chronic respiratory conditions, including chronic obstructive pulmonary disease, chronic bronchial disease, respiratory failure, pneumocardial disease, or acute pneumonia within 3 months before fracture), chronic renal disease (pre-existing known renal disease but not elevated urea without a diagnosis of a renal condition), chronic liver disease (hepatitis, liver cirrhosis, but not liver cancer), diabetes, rheumatic disease, and malignancy. The outcomes examined in this study were 1- and 12-month mortality, postoperative complications, and LOS. We divided the postoperative complications into cardiovascular and cerebrovascular diseases and infectious diseases. The infectious diseases comprised superficial infection, deep infection, pneumonia, urinary tract infection, and decubitus pressure ulcers. All patients were contacted by 1 of 2 trained interviewers to obtain follow-up information at 12 months after fracture or until death. If a patient was not available, a family member or caregiver was interviewed.

Patients were grouped into tertiles for mean FPG (<8.72, 8.72–10.37, and >10.37 mmol/L) and CV of FPG (<0.082, 0.082–0.203, and >0.203). All statistical analyses were performed using IBM SPSS Statistics for Windows, Version 19.0. (Armonk, NY: IBM Corp). The chi-square statistic was used to compare the proportional results. The mean and standard deviation were calculated for continuous data, which were analyzed using the unpaired *t* test with Welch correction for unequal variances. Pearson correlation coefficient (r^2^) was used to measure the strength of a linear association between mean FPG and CV of FPG. Finally, Cox regression was undertaken to estimate the impact of GV on the mortality of patients. *P* values of .05 or less were considered significant.

## 3 Results

**Figure 1:**
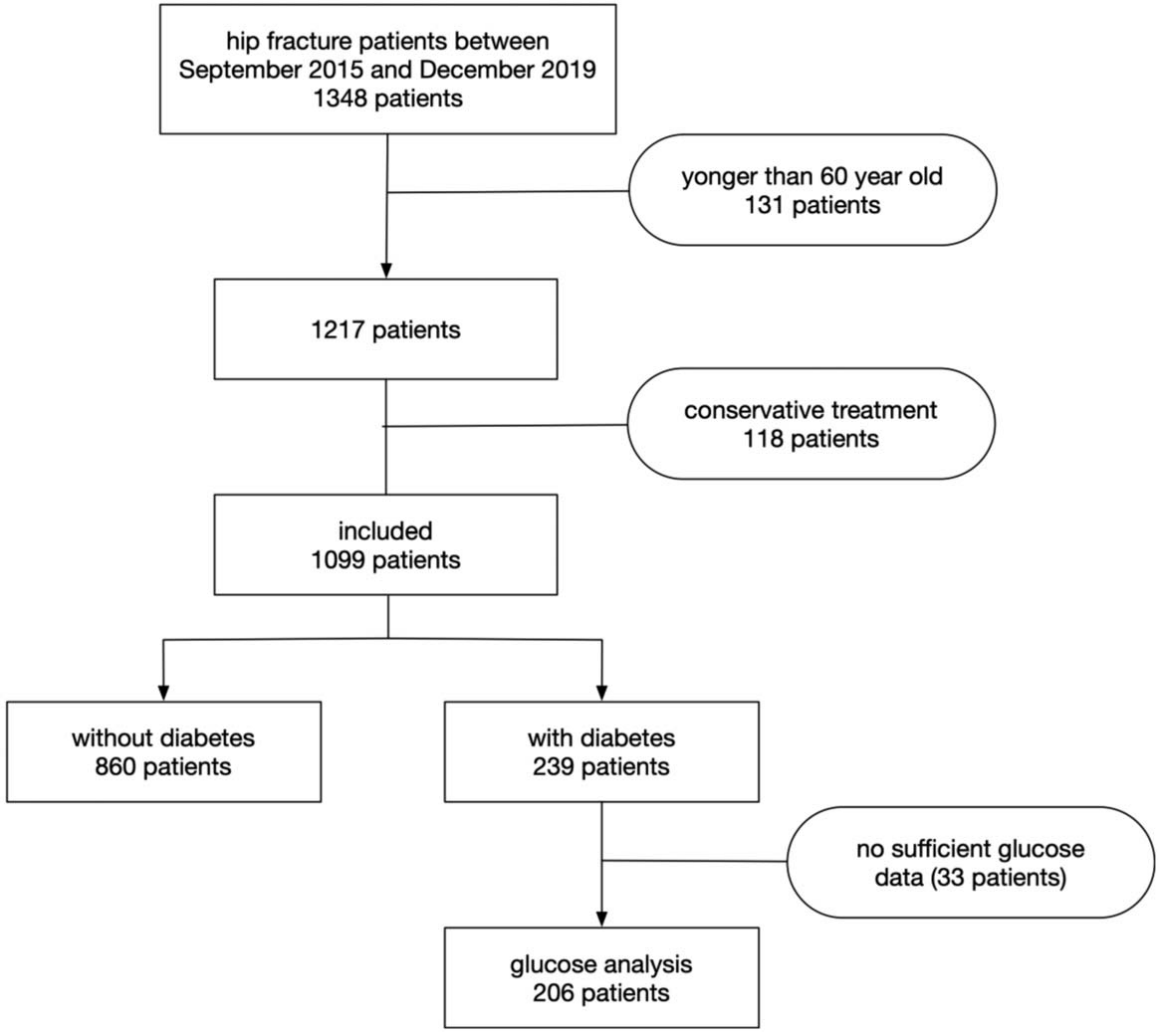
Flowchart of included patients and data analysis in this study.

In this study, 1348 patients with hip fractures were admitted to our hospital between September 2015 and December 2019. In total, 131 (9.7%) patients younger than 60 years old were excluded from this study. For clinical consideration and evaluation, 118 (8.7%) patients were treated conservatively because of their high risks of anesthesia- and surgery-related complications. Thus, 1099 patients met the criteria for inclusion in this study. These demographic characteristics are summarized in Table 1. There were 612 (55.7%) patients diagnosed with femur neck fracture and 487 (44.3%) patients diagnosed with intertrochanteric fracture. The mean age of the patients was 75.1 ± 10.3 years, and 694 (63.1%) patients were women. Of these, 239 (21.7%) patients were diagnosed with diabetes, and the median duration of diabetes was 10 years. However, we could not divide the patients into type 1 and type 2 diabetes groups because of a lack of specific data. Unfortunately, 33 (13.8%) patients had no sufficient glucose data during the perioperative period. All patients entered/enrolled in this study are accounted for in Figure 1.

**Table 1:** Pre-operative characteristics and postoperative outcomes between non-diabetes and diabetes patients.

	Non-diabetes	Diabetes	*P* value
N	860	239	
Gender (female)	534 (62%)	161 (67.4%)	.13
Age (yrs)	74.9 ± 10.6	75.5 ± 8.7	.421
BMI (kg/m^2^)	22.5 ± 3.9	23.8 ± 3.5	<.001
Type of fracture (intracapsular)	490 (56.9%)	122 (51.0%)	.106
Number of comorbidities^∗^			<.001
0	344 (40.0%)	50 (20.9%)	
1	275 (31.9%)	84 (35.1%)	
2	150 (17.4%)	64 (26.8%)	
3+	92 (10.7%)	41 (17.2%)	
Time to surgery (d)	4 (2, 6)	5 (3, 7)	.001
LOS (d)	12 (9, 15)	12 (10, 16)	.029
Postoperative complications	70 (8.1%)	30 (12.6%)	.036
Cardiovascular or cerebrovascular complications	24 (2.8%)	13 (5.4%)	.045
Infectious complications	63 (7.3%)	24 (10.0%)	.169
Mortality within 1 mo	18 (2.7%)	12 (5.5%)	.052
Mortality within 12 mos	57 (8.7%)	33 (15.1%)	.006

BMI = body mass index, LOS = hospital length of stay.

∗ The number of comorbidities is not included diabetes.

The patient's characteristics, comorbidities, fracture patterns, and postoperative outcomes are detailed in Table 1. Patients with diabetes had a higher body mass index than those without diabetes (*P* < .001). Approximately 79.1% of all patients with diabetes had at least 1 comorbidity, as opposed to 60% of those without diabetes (*P* < .001). There were no differences between patients with and without diabetes regarding age, sex, and fracture patterns. Patients with diabetes were found to have a higher incidence of postoperative complications (12.6% vs 8.1%; *P* = .036). Subgroup analysis revealed that infectious diseases were more prevalent in patients with diabetes (5.4% vs 2.8%; *P* = .045). However, when comparing the incidence of cardiovascular and cerebrovascular diseases, there were no differences between the 2 groups (10.0% vs 7.3%; *P* = .169). The mortality rate was higher in patients with diabetes at both 1 (5.5% vs 2.7%; *P* = .052) and 12 months (15.1% vs 8.7%; *P* = .006) postoperatively.

Table 2 details the patients’ characteristics and outcomes among the tertiles of both mean FPG and CV of FPG. The overall relationship between mean FPG and CV of FPG was relatively weak (r^2^ = 0.066, *P* = .349). There were no differences among the tertiles of both mean FPG and CV of FPG concerning age, sex, and fracture patterns. Postoperative complications and mortality did not differ among the tertiles of mean FPG. Similar results were observed for the tertiles of GV; however, it should be noted that the 1-month mortality rate was higher in 2^nd^ and 3^rd^ tertiles than the 1^st^ tertiles of GV.

**Table 2:** Demographic and clinical characteristics of mean FPG and of the CV of FPG in diabetic patients.

	Tertiles of mean FPG				Tertiles of GV			
	<8.72	8.72–10.37	>10.37	*P* value	<0.082	0.082–0.203	>0.203	*P* value
Number	68	69	69	NA	68	69	69	NA
Age (yrs)	76.3 ± 6.6	77.6 ± 8.5	75.1 ± 7.9	.181	77.0 ± 6.7	74.6 ± 7.9	77.3 ± 8.7	.094
Gender (female)	47 (69.1%)	52 (74.3%)	44 (63.8%)	.407	45 (66.2%)	50 (72.5%)	47 (68.1%)	.717
BMI (kg/m^2^)	23.3 ± 3.8	24.4 ± 3.4	23.8 ± 3.6	.189	23.9 ± 3.1	24.3 ± 3.9	23.2 ± 3.6	.209
Mean FPG	7.9 ± 0.58	9.5 ± 0.51	11.9 ± 1.69	<.001	9.7 ± 2.4	9.7 ± 1.5	10.0 ± 2.0	.462
GV of FPG	0.236 ± 0.096	0.262 ± 0.088	0.262 ± 0.093	.173	0.159 ± 0.028	0.242 ± 0.021	0.358 ± 0.068	<.001
Type of fracture (intracapsular)	32 (47.1%)	40 (57.1%)	30 (43.5%)	.247	28 (41.2%)	36 (52.2%)	38 (55.1%)	.23
Time to surgery (d)	6 (4, 8)	5 (3, 7)	4 (3, 6)	.087	5 (3, 6)	5 (3, 7)	5 (3, 7)	.816
LOS (d)	12 (11, 17)	12 (9, 18)	12 (9, 14)	.995	12 (10, 16)	13 (10, 18)	12 (9, 17)	.308
Postoperative complications	9 (13.2%)	12 (17.1%)	7 (10.1%)	.481	8 (11.8%)	9 (13.0%)	11 (15.9%)	.765
Cardiovascular or cerebrovascular complications	7 (10.3%)	9 (12.9%)	7 (10.1%)	.849	5 (7.4%)	8 (11.6%)	10 (14.5%)	.411
Infectious complications	4 (5.9%)	5 (7.1%)	3 (4.3%)	.779	4 (5.9%)	5 (7.2%)	3 (4.3%)	.768
Mortality within 1 mo	3 (4.8%)	5 (8.1%)	5 (7.7%)	.724	1 (1.6%)	3 (4.8%)	9 (13.8%)	.018
Mortality within 12 mos	8 (12.7%)	9 (14.5%)	11 (16.9%)	.795	5 (8.1%)	11 (17.7%)	12 (18.5%)	.188

BMI = body mass index, CV = coefficient of variation, FPG = fasting plasma glucose, GV = glucose variability, LOS = hospital length of stay.

Multivariate survival analysis performed using the Cox regression model (Table 3) revealed that both the mean FPG and CV of FPG were independent predictors of mortality within 1 month postoperatively, and GV, but not mean FPG, was an independent predictor of mortality within 12 months postoperatively. In particular, patients in the 2^nd^ and 3^rd^ tertiles of GV had an 80% higher risk of all-cause mortality.

**Table 3:** Multivariate Cox regression analysis of all variables for 1 and 12 months mortality.

	1 mo		12 mos	
	HR (95% CI)	*P* value	HR (95% CI)	*P* value
Sex	0.684 (0.146, 2.207)	.630	1.038 (0.392, 2.753)	.940
Age	1.124 (1.006, 1.257)	.039	1.052 (0.990, 1.118)	.100
Number of comorbidities^∗^	1.277 (0.597, 2.729)	.529	1.207 (0.800, 1.821)	.371
Type of fracture (intracapsular)	4.451 (1.059, 18.741)	.042	0.902 (0.374, 2.175)	.819
Mean FPG				
2^nd^ tertile vs 1^st^ tertile	5.516 (0.791, 38.449)	.085	1.448 (0.527, 3.979)	.473
3^rd^ tertile vs 1^st^ tertile	4.245 (0.508, 35.465)	.182	1.463 (0.564, 3.797)	.434
CV of FPG				
2^nd^ tertile vs 1^st^ tertile	12.812 (1.013, 162.023)	.049	2.852 (0.959, 8.484)	.060
3^rd^ tertile vs 1^st^ tertile	8.308 (0.968, 71.304)	.054	2.025 (0.706, 5.812)	.190

95% CI = 95% confidence interval, CV = coefficient of variation, FPG = fasting plasma glucose, HR = hazard ratios.

∗ The number of comorbidities is not included diabetes.

## 4 Discussion

A previous study and guidelines suggested that well-controlled perioperative glucose could improve the outcomes of patients during surgery.^[19,20]^ In addition, some studies indicated that GV could be a predictor of diabetic complications, independent of HbA1c levels, in patients with diabetes, and better daily control of blood glucose excursions may reduce the risk of these complications.^[21]^ As far as we know, this study was the first to compare outcomes among tertiles of GV during the perioperative period for patients with hip fracture.

Consistent with other studies,^[2,3,7]^ we found that patients with diabetes have an increased risk of mortality after hip fracture. In addition to mortality within 1 month postoperatively, patients with diabetes had a much higher risk of 12-month mortality. We also compared postoperative complications between patients with and without diabetes. All complications, especially infectious complications, were more common in patients with diabetes. Therefore, to decrease complications and mortality rates, medical care in the postoperative period should be optimized in patients with diabetes who experience hip fracture.^[22,23]^

Recently, 1 system review reported that higher GV was strongly associated with a higher risk of complications.^[16]^ However, we could not find any differences among the tertiles of mean FPG and GV for postoperative complications.

Although mean FPG was an essential target for the perioperative management of diabetes, our data proved that the different tertiles of mean FPG were correlated with insignificant mortality. Despite this, the mortality rate was higher across the tertiles of GV. The patients in the lowest GV tertile exhibited a much lower mortality rate than in the other groups. We should note that the mortality rate in the middle and upper tertiles of GV was similar to each other within 1 year. This result suggests the existence of a threshold for long-term mortality.

Multivariate analysis demonstrated that age, intertrochanteric fracture, and higher variability of FPG were independent predictors of mortality within 1 month postoperatively for patients with diabetes who experience hip fracture. However, these risk factors were not independently associated with 12-month mortality. This does not necessarily mean that the severity of hyperglycemia was not crucial for predicting the outcome in diabetes, but it indicates that the predictive value of mean FPG was lower than that of GV. Indeed, our data suggest that GV might be more reliable than mean FPG in assessing the relationship between glucose control and survival.

However, there were several limitations to our study. First, we could not divide patients with diabetes into type 1 and type 2 diabetes groups because this information was not collected at the time of admission. As glucose management may differentiate between type 1 and type 2 diabetes, GV could be significantly different. Second, due to the retrospective design, we were unable to obtain the hemoglobin A1c which represents the average level of blood glucose over the past 2 to 3 months. Furthermore, because we lost to follow-up more diabetic than non-diabetic patients after 1 year (6% more losses in the diabetes group), we cannot rule out a bias on the result of the multivariate survival analysis.

## 5 Conclusion

In conclusion, the results of this study suggest that patients with diabetes who suffered hip fractures were more likely to experience infectious complications and mortality postoperatively. Most importantly, this study indicated that GV might be useful for evaluating glucose control to predict survival in patients with diabetes who suffered hip fractures.

## Acknowledgments

No potential conflicts of interest relevant to this article were reported. We thank Joe Barber Jr., PhD, from Liwen Bianji (Edanz) (www.liwenbianji.cn/), for editing the English text of a draft of this manuscript. We thank Feifei Zhao and Lu Jin for helping us build the Hip Fracture database.

## Author contributions

**Conceptualization:** Anhua Long, Yakui Zhang, Dacheng Han.

**Data curation:** Anhua Long, Xuefei Wang.

**Investigation:** Xuefei Wang, Yakui Zhang.

**Methodology:** Yakui Zhang.

**Resources:** Xuefei Wang.

**Software:** Zongyan Xie.

**Supervision:** Zongyan Xie.

**Validation:** Zongyan Xie.

**Writing – original draft:** Anhua Long.

**Writing – review & editing:** Yakui Zhang, Dacheng Han.
